# Supplementary material for: Emergency department visits among patients transported by law enforcement officers
Source: PLoS One. 2021 Jan 13;16(1):e0244679. doi: 10.1371/journal.pone.0244679 (PMC7806121; doi:10.1371/journal.pone.0244679)
Supplement: S1 Fig — The figure demonstrates that the steep increase in MH-related ED transports begins approximately 4 months following the transition from ICD-9 to ICD-10 suggesting that the increase is not simply an artifact of the transition. (PDF) [file pone.0244679.s001.pdf]

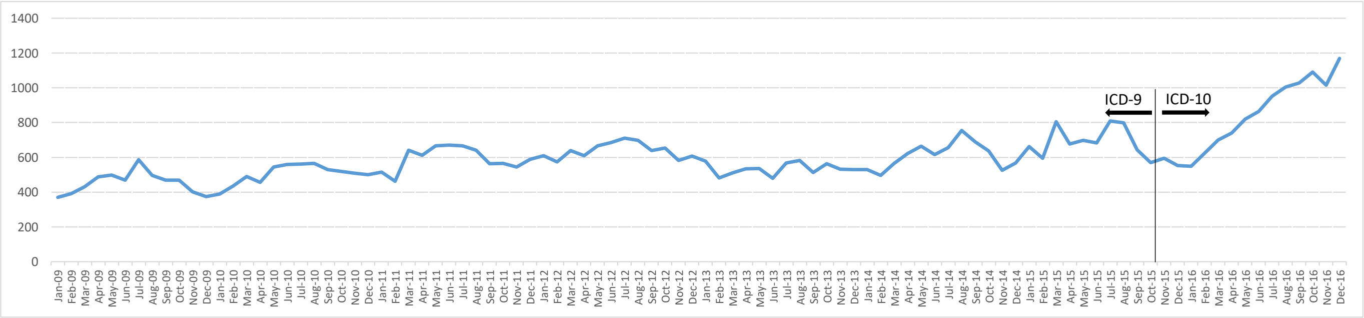

S1 Fig. Number of mental health-related EDs transports by law enforcement, North Carolina, 2009 - 2016. The figure demonstrates that the steep increase in MH-related ED transports begins approximately 4 months following the transition from ICD-9 to ICD-10 suggesting that the increase is not simply an artifact of the transition
